# The overlap of anti‐Black and anti‐protest rhetoric: How far‐right political commentators preserve anti‐Black racist stereotypes in the context of Black Lives Matter debates

**DOI:** 10.1111/bjso.70046

**Published:** 2026-01-26

**Authors:** Alexander Hunt, Mirko Demasi, Simon Goodman

**Affiliations:** ^1^ School of Computing Sciences University of East Anglia Norwich UK; ^2^ School of Humanities and Social Sciences Leeds Beckett University Leeds UK; ^3^ School of Applied Social Sciences De Montfort University Leicester UK

**Keywords:** anti‐Black racism, anti‐protest, Black Lives Matter, categories, discursive and rhetorical psychology, ideology

## Abstract

Research has shown that speakers opposing political demonstrations can pathologize protesters campaigning against racial prejudice in order to justify racialized police profiling and brutality. This paper builds on these insights by exploring how right‐wing political commentators reinforce the racist stereotype of violent Black people when discussing protests and police brutality in Black Lives Matter (BLM) debates. The dataset includes two debates drawn from *Conservative Talk Radio* and *The Candace Owen Show*, where issues concerning anti‐Black racism in the United States were discussed—including racialized police brutality and BLM demonstrations. Using discursive and rhetorical psychology, we show how far‐right commentators managed their (arguably racist) identities by employing ‘rioter’ categories against the BLM movement. We demonstrate that far‐right commentators used anti‐protest rhetoric and anti‐Black racist tropes to portray BLM activists as uncivilized and violent rioters. Doing so portrayed the BLM movement as using anti‐racism as an ulterior motive to enact violence which also downplayed racialized police brutality. This study shows how anti‐protest rhetoric and anti‐Black stereotypes overlap when right‐wing speakers undermine attempts to challenge systemic racism. Black people and protesters are discriminated against in similar ways; both are characterized as violent and uncivilized when they mobilize against structural oppression and inequality.

## INTRODUCTION

The present study explores how right‐wing US political commentators rhetorically undermine Black Lives Matter (BLM)‐related protests opposing anti‐Black racism. Despite the civil rights movement's success in overturning the Jim Crow segregation laws, racial prejudice against the Black community in the United States remains an institutional issue. Of particular relevance to the present study are reports demonstrating a disproportionately high number of Black people who are physically brutalized or shot by police officers. Since 2012, the backlash to these incidents led to the initiation of the BLM movement, indicating a broad disapproval of racial prejudice against the Black community. The origins of the BLM movement trace back to March 2012—when a neighbourhood watch volunteer, George Zimmerman, shot and killed 17‐year‐old African American Trayvon Martin after the police were called on him for suspicious behaviour.

Although accounts later indicated that Martin was heading to a convenience store, Zimmerman was not charged as he appealed to Florida's ‘stand your ground’ law—leading to the jury determining him as not guilty. After the verdict, Patrisse Cullors, Alicia Garza and Opal Tometi inspired the BLM movement through various social media posts under #BlackLivesMatter (Rickford, 2016). Despite the success of its influence across social media platforms, BLM became controversial when right‐wing commentators opposed the movement (Hoffman et al., 2016). Critics of BLM particularly sought to refute claims that the treatment of the Black community stems from institutional racism. The BLM movement typically manifested through demonstrations and protests to bring justice and heal the Black community. These efforts were, however, obstructed by right‐wing political commentators who used their platform to criticize BLM demonstrations for seeking to violently dismantle civilized society. This shows that the use of conflicting explanations of BLM‐related crowd events between right‐wing political commentators and anti‐racist activists can be seen as rhetorical strategies to either challenge or support the movement.

Right‐wing political commentators also sought to exonerate cases of violent police behaviour against Black people from racial prejudice. Although Black people are more likely to be stopped, targeted and assaulted by law enforcement (e.g. ACLU, 2020; Kishi & Jones, 2020), demonstrations of social justice generally escalate into riots because of police action as opposed to crowd members behaving disorderly (e.g. Stott et al., 2018). Despite this, right‐wing commentators sought to implicate Black people as instigators of violent riots—thus, reinforcing anti‐Black stereotypes and outdated views on crowds (e.g. Norman, 2020). One way to deconstruct this issue is to consider how right‐wing commentators rhetorically denigrate BLM‐related protests in order to reinforce the racist stereotype of violent Black rioters. Previous research by Perkins et al. (2019) explored anti‐Black racism by showing how Black speakers negotiated responsibility and agency when discussing their racialized experiences. Building on this work, our aims concern how speakers invoke and draw on anti‐protest positions with anti‐Black stereotypes to preserve structural inequalities in the United States. We will first outline the discursive psychological approach to racism to establish the theoretical foundation of our study.

### Discursive psychological approach to prejudice

Potter and Wetherell (1987) developed the discursive approach to social psychology as a critique of how scholars traditionally explained psychological concepts in relation to talk and text (see also Edwards & Potter, 1992). Rather than offering windows into the mind, discursive psychology (DP) considers how talk and text are used to construct social reality to achieve social actions in specific social contexts. Since its development, DP has shown a capacity to engage with psychological research in a novel way by showing how speakers invoke psychological concepts like memories, attitudes and emotions in discourse to accomplish social actions (Edwards & Potter, 2005). For the purpose of the present study, we build on DP research that engaged with the notion of prejudice (e.g. Augoustinos & Every, 2007; Tileagă, 2007; Wetherell & Potter, 1992). Rather than understanding prejudice as an abstracted cognition located within the minds of irrational people, DP provides empirical insights into how it functions as a dynamic process enacted in social interaction. The DP perspective thus locates prejudice in action rather than irrational/faulty thought processes (Billig, 2002).

This approach encourages us to consider discourse as the means by which social life unfolds (Demasi, 2022)—thus, allowing social psychologists to fully consider how prejudice is ‘lived’ (Billig et al., 1988). In this sense, DP proposes that prejudice manifests through language itself when speakers draw on shared common‐sense ideological assumptions and values that organize social life (Durrheim, 2016). Billig (1978) notes that there is a norm against being seen as prejudiced to the extent that extreme forms of racism come across as unreasonable (see also Billig et al., 1988). This means that arguably prejudicial comments are required to be carefully expressed as neutral and rational to avoid potential criticisms (Augoustinos & Every, 2007; Durrheim et al., 2016)—a point equally delicate when it comes to making accusations of prejudice (Gibson, 2021; Goodman, 2010, 2014). As such, ‘hearably prejudiced talk is an accountable matter for those producing it and for those who resist it. Viewing prejudice as such enables one to study it as a flexible, occasioned and rhetorically oriented resource’ (Burke & Demasi, 2021, p. 206).

To demonstrate and explain the rhetorically complex and delicate nature of prejudice, we can investigate mundane everyday language (Edwards, 2003). Identities and categories are crucial features of everyday social interaction where prejudiced discourse unfolds. For example, particular identities can be paired with an expletive to mark them as prejudiced (Burke & Demasi, 2021; Stokoe & Edwards, 2007). Of particular analytic interest for us, however, in the present study are the more subtle expressions of prejudice. Tileagă (2007) demonstrated how placing a category of people outside a moral boundary allows speakers to dehumanize certain groups without resorting to hostile, overtly racist language. Subtle expressions of prejudice can be understood as a means of distancing a group from dignity and civility without explicit expressions of hatred (Tileagă, 2015). Rather than understanding these descriptions as neutral reports of social life, the conventions of DP indicate that people use descriptions to perform identity and category work (Edwards & Potter, 1992). It is thus important to look at how speakers talk about protests against racism—especially when superficial politeness provides a rhetorical means of enacting dehumanization in practice (Burke & Demasi, 2021).

### Social justice movements and the language of racism

When investigating protests, particular groups of social psychologists typically adopt a social identity framework to explore how crowds mobilize against inequalities and prejudices including anti‐Black racism. For example, Drury et al. (2020) argued that the spread of the 2011 riots in England was caused by hostility towards the police and feelings of institutional alienation. In addition to the police profiling and conducting high levels of searches against young Black men, the then Conservative government's economic system of austerity left people feeling alienated (Gentleman, 2019). Crowds mobilized in London to protest injustices such as racial inequality and socio‐economic disparity, which in turn led to further riots. Similarly, Uluğ and Tropp (2021) investigated instances where racial prejudice against Black people motivated White people to engage in collective action for racial equality. The authors demonstrated that when racial groups witnessed prejudice, it became difficult for them to deny the existence of racism. While research grounded in social identity theory demonstrates that collective action is a response to racial prejudice (see also Brown et al., 2022; Ferguson et al., 2019), speakers can use rhetoric to frame such protests as unreasonable.

Drury (2002) particularly demonstrated that classical crowd characterizations including ‘mob rule’, ‘moral panic’ and ‘communal hysteria’ (see Le Bon, 1947) rhetorically delegitimize collective action that promotes social justice (see also Stott & Drury, 2017). Similarly, Potter and Reicher (1987) illustrated that news reporters and police officers characterized the 1980 crowd events of St Paul's as ‘destructive riots’ despite participants of these protests claiming that they sought to protect their community from racism. Wetherell and Potter (1992) were particularly instrumental in illustrating how the language of racial prejudice is used to discredit minority groups participating in protests and activism. They demonstrated how New Zealanders of European descent provided contrasting rhetorical explanations of why Māori minority groups participated in protests. While one side recognized activism as essential to social change, others described Māori people's motives for protesting as disingenuous and hypocritical by calling doubt into their marginalized group status. As such, these contrasting positions of crowd behaviour can be understood as rhetorical issues which speakers negotiate for ideological purposes (e.g. Reicher & Hopkins, 2001). The ideological function concerning the language of prejudice particularly preserves and legitimizes racial prejudice by undermining movements against race‐related social injustices.

The way in which the language of prejudice undermines protest movements advocating social justice particularly has historical and institutional implications for the preservation and reinforcement of anti‐Black racist stereotypes. As an example, van Dijk (1992a) highlighted that the British tabloid press predominantly talked about Black men in relation to their reports on urban disturbances. Black men were typically characterized as violent rioters because of their propensity to criminal behaviour—as opposed to their feelings of anger and frustration of being systemically discriminated against. By portraying Black people as violent criminals, news media legitimize and reinforce anti‐Black racist stereotypes of uncivilized and violent groups (e.g. Helg, 2000; Smiley & Fakunle, 2018; Welch, 2007). This characterization of Black people can be arguably tied back to our discussion of how crowds opposed to racial prejudice were rhetorically discredited as violent and uncivilized. Given that particular participants of the Black Lives Matter movement can encompass the combined identity of ‘Black protesters’, there is potential to empirically explore the ideological implications of ‘anti‐Black’ and ‘anti‐protest’ rhetoric overlapping in discourse.

### DP perspectives on BLM and police violence

While some DP studies addressed BLM, they explored it through online contexts unrelated to accounts of crowd behaviour. Goodman et al. (2023) notably explored online debates about BLM by showing alternative ‘lives matter’ slogans. They particularly demonstrated how social media posters used the ‘All‐Lives Matter’ slogan to undermine the anti‐racist message of BLM—thus serving to distance the issue of racism from the debate. While West et al. (2021) showed how those that score highly on measures of racism are more likely to favour All‐Lives Matter over BLM, Goodman et al. (2024) demonstrated that Twitter users criticized the All‐Lives Matter slogan for being racist. In building upon these discursive and rhetorical psychological studies, we paid particular attention to how BLM demonstrations mobilized as a response to disproportionately high police brutality against Black people. As such, BLM debates where speakers account for police and protester actions are ideal discursive and rhetorical contexts to explore the combined uses of anti‐Black and anti‐protest rhetoric. While previous discursive studies explored accounts of racialized police violence, their focus was unrelated to the overlap of anti‐Black stereotypes and anti‐protest rhetoric.

This discursive work notably focused on how speakers manage issues of accountability when talking about police violence. Wetherell and Potter (1989) in particular interviewed New Zealanders of European descent about political issues concerning controversial incidents that occurred during the 1981 Springbok Rugby tour. Anti‐apartheid campaigners mobilized to protest during the matches and the police physically assaulted them during one of the demonstrations which was reported as a ‘riot’. The interviewees were asked whether these acts of police brutality were a justifiable recourse against the protesters. Those who justified police brutality argued that law enforcement officers were merely fulfilling their duty and that violence was a natural human response to the anti‐apartheid protesters who provoked them. Shrikant and Sambaraju (2021) on the other hand demonstrated how news media used category formulations when reporting US police shootings of Black people. The news reports used a categorical pairing by emphasizing that the victims were both Black and unarmed to frame the shootings as unwarranted enough to legitimize police action as racialized. Sambaraju (2025) similarly illustrated that speakers legitimized the prevalence of institutional anti‐Black racism in India by arguing that the police profiled and assaulted Black Indians.

### Aims and rationale

Synthesizing the previous research outlined above highlights that protesters and Black people are frequently discussed as victims of police brutality—indicating that both groups are subject to similar forms of structural inequality. In the present study, we aim to expand upon the discussions outlined throughout this introduction by demonstrating how right‐wing activists justify racialized police brutality in the United States to undermine BLM‐related protests. Although previous crowd psychology research demonstrates how protesters mobilize to challenge racial prejudice, right‐wing political activists undermine these efforts by using their rhetorical skills to distance police violence from racism. As such, right‐wing activists rhetorically legitimize and reinforce anti‐Black racist stereotypes in the United States by presenting BLM protesters as predisposed to violent criminal behaviour—thus, preserving systemic racism by association. By exploring how these accounts are discursively worked up, we aim to deconstruct the common‐sense assumption which associates participants of BLM demonstrations with violent rioters. In doing so, we also aim to build on discursive psychological approaches to prejudice by considering the ideological overlap concerning anti‐protest rhetorical strategies and anti‐Black stereotypes.

## METHODS AND DATA

The dataset comprised two debate recordings (total duration: 3 h 26 min) of discussions concerning systemic racism against the Black community in the United States. The first debate is titled ‘Race in America’ which was hosted on KTTH 770 conservative talk radio in April 2015. The debate comprised four panellists and of particular interest to our aims were two conservative commentators including the former state representative candidate Monique Valenzuela Trudnowski and Ben Shapiro, the co‐founder of ‘The Daily Wire’. The debate covered issues relating to anti‐Black racism in the United States including the BLM movement which became influential following police shootings of Black teenagers including Michael Brown, Eric Garner and Freddie Gray. These incidents sparked the 2015 Baltimore protests of which the panel members discussed to contest the legitimacy of anti‐racist movements. The second debate was an episode of a podcast hosted by conservative activist and co‐founder of the BLEXIT foundation Candace Owens. She argued against Hawk Newsome, the co‐founder of the New York chapter of BLM, to downplay the severity of anti‐Black racism in the United States.

We drew on the conventions of discursive (Edwards & Potter, 1992; Potter, 1996; Potter & Wetherell, 1987) and rhetorical psychology (Billig, 1987, 1991; Billig et al., 1988) to analyse this dataset. This framework allowed us to effectively explore how the commentators in the debates constructed versions of events concerning BLM‐related crowd events, and police shootings of Black people, in ways that oriented towards their argumentative positions. To highlight these contrasting arguments, we focused on how speakers used discursive categories to describe and indicate their stances on these events. Edwards (1997) demonstrated that when people recount their version of events, they categorize them in particular ways to achieve social actions like managing accountability or undermining and justifying positions. As discussed, Potter and Reicher (1987) indicated that the descriptive ‘rioter’ is a discursive category that associates crowd events with violence and destruction. We therefore extended their findings to our analytical framework to demonstrate how anti‐BLM positions, and anti‐Black racism by association, are justified through negative portrayals of crowds. We therefore explored how right‐wing commentators used the ‘rioter’ category to reinforce anti‐Black stereotypes that portray Black protesters as uncivilized and prone to violence.

We combined our discursive framework with rhetorical psychology—Billig (1987) claimed that the way in which speakers debate contrasting positions reflects wider ideological assumptions concerning particular societal issues. This approach notably demonstrated the broader ideological implications underlying the overlap of anti‐protest rhetoric and anti‐Black stereotypes when right‐wing commentators criticized the BLM movement. As discussed, discursive and rhetorical psychological research highlights that speakers avoid and deny prejudiced identities when navigating race talk (Augoustinos & Every, 2007; Condor et al., 2006; Wetherell & Potter, 1992). When right‐wing commentators opposed the BLM movement, they sought to deny that they were racist. As discussed with Billig's (1978) proposition, speakers who express overtly racist remarks in institutional settings risk looking irrational to the extent of harming the credibility of their arguments. Given that the two debates were broadcast for public viewing, it was important for influential speakers like right‐wing commentators to depict their anti‐BLM stance as reasonable and distanced from racism. We considered how the right‐wing speakers used various rhetorical strategies to justify describing BLM protests as violent riots, while indicating that police shootings of Black people were unrelated to racism. The combined framework of discursive and rhetorical psychology highlighted how anti‐BLM rhetoric constructed protests against racial inequality as unreasonable to the extent of preserving US anti‐Black tropes.

## ANALYSIS

Our analysis is divided into two subsections, each highlighting distinct discursive and rhetorical strategies that were used to preserve anti‐Black racism in the United States. The first subsection focuses on how right‐wing political commentators undermined the legitimacy of BLM by deracializing police brutality against Black people. The second meanwhile explores the discursive categories which the commentators employed to characterize BLM‐related crowd events as violent.

### Justification of racialized police brutality against the Black community

In the dataset, right‐wing commentators typically distanced the notion of civility from the Black community to frame police brutality as a justifiable response. One rhetorical strategy they used to justify this construction was to claim that Black people were inherently disobedient towards authority to the extent that police violence was made hearably necessary. As an example, consider the following extract from the KTTH 770 conservative talk radio—‘Race in America’ debate where Ben Shapiro justifies why the police shot Michael Brown and Eric Garner—two Black teenagers. 

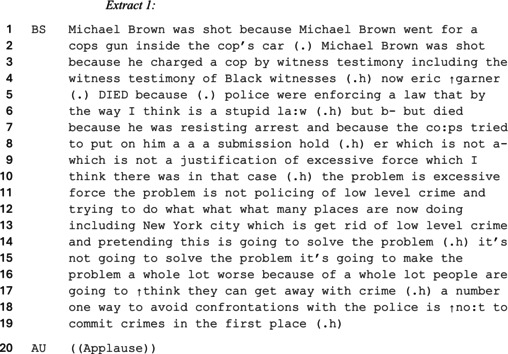

In this extract, Shapiro constructs a deracialized account (Augoustinos & Every, 2007) to shift blame for police brutality away from racism and towards the behaviour of Black people encountered by police. Shapiro's closing statement particularly frames Black people themselves as accountable for their own problems with the police by committing crimes in the first place. He legitimizes his deracialized argument by corroborating other Black people as reliable witnesses of Michael Brown charging a police officer—thus, managing the witnesses and his own stake about the potential racist nature of the shooting (Potter, 1996). Shapiro constructs racism and excessive force as two distinct forms of police enforcement when describing Eric Garner's interaction with the police. By claiming that the incident was a matter of excessive force, he frames himself as acknowledging the existence of police brutality without conceding that racism is attributed to the deaths of Black people. Shapiro depicts himself as distinguishing between racism and excessive force to justify his claim that Black people are killed by police due to their violent behaviour—rather than because of police conduct itself.

Shapiro uses a common‐sense argument—evidenced by the support of the applause on line 20 (Billig & Marinho, 2017)—which implies that people are safe from police encounters if they are innocent. Shapiro therefore bolsters his position that, regardless of whether excessive force is used, encounters with the police only happen to those who break the law. As such, Shapiro uses a just‐world argument to treat police brutality as a form of excessive force unrelated to racism (Goodman & Carr, 2017). Shapiro thus frames himself as reasonable for claiming that Black victims of excessive police force are responsible for their actions and criminal tendencies. In addition to framing excessive police force as more relevant than racism, Shapiro seeks to absolve the policing of low‐level crime from culpability. Doing so continues to deracialize police action by constructing a common‐sense argument indicating that anyone who breaks the law—regardless of their race and degree of committed crime—should be punished by the police to preserve law and order (Billig, 1987). Shapiro's argument preserves anti‐Black racism by strengthening the association between Black people and crime, while couching this in deracialized terms.

In other cases where right‐wing commentators resisted claims (implied or actual) of police brutality, they also argued that the media exaggerated the extent to which Black people were profiled and targeted by law enforcement. This is evident in the next extract from the ‘Candace Owens Show’ where Owens frames news reports concerning racialized police brutality as conspiracies made to emotionally manipulate Black voters into supporting the Democratic party. 

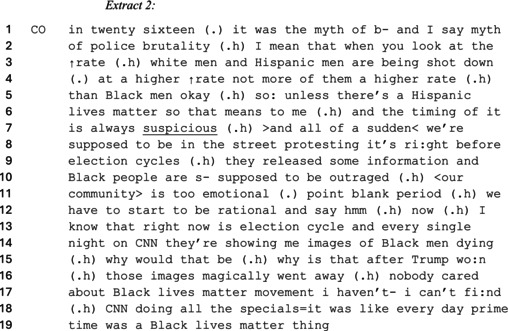

Owens categorizes 2016 news reports of police brutality as a ‘myth’ (line 1) to delegitimize claims that the Black community is disproportionately targeted by the police. She portrays police brutality as a matter inflicted upon white and Hispanic people ‘at a higher rate than Black men’ (lines 4). The words ‘if you look at the rates which…’ (lines 2–3) suggest a visual metaphor, reifying Owens' position (Demasi, 2023). By undercutting the factuality of a core BLM point on line 7, Owens posits that there is a conspiracy behind the reports of police brutality against the Black community (Byford & Billig, 2001). By rhetorically asserting that ‘unless there is a Hispanic lives matter’ (lines 5–6), Owens criticizes media support for BLM as insincere for handpicking or manipulating information to suit the agenda of an argument (Billig & Marinho, 2014, 2025). She thus presents the timing of mainstream media coverage of BLM as ‘suspicious’ (line 7) to strengthen the likelihood of a hidden agenda. Owens portrays herself as having deduced this to justify accusing CNN (a left‐leaning news outlet) of manipulating information for political reasons instead of a legitimate concern.

By claiming that ‘after Trump won those images magically went away’ (line 15–16), Owens legitimizes her argument that the reports of racialized police brutality were politicized to win votes. Owens claims that reports about the Black community being targeted by police brutality stopped after the 2016 US presidential election cycle to undermine the BLM movement as a political pawn. She counterclaims that reports of racialized police brutality against the Black community were ‘supposed to outrage’ (line 10) them. Owens aligns her position with the interest of the Black community by using inclusive pronouns such as ‘we’ and ‘our’ (Goffman, 1979) to position and categorically entitle herself as a member of the Black community (Shrikant & Sambaraju, 2024). Constructing her identity as a Black person mitigates the racist connotations of claiming that the Black ‘community is too emotional’ (line 11). Owens thus indicates that the Black community is lacking, if only temporarily, the faculties for clear and rational thinking. Her version of critical thinking suggests that rational people would see the timing of BLM‐related media reports as tainted by the manipulation of a left‐wing news outlet—thus distancing rationality from BLM advocacy. The psychological makeup of a decent, rational person, according to Owens, does not support BLM.

We demonstrated in this first subsection how right‐wing political commentators preserved anti‐Black racism by delegitimizing the racialization of police violence. Although right‐wing commentators did not deny the existence of police violence, they treated their actions as irrelevant to racism to justify opposing BLM. They also raised the possibility of a political agenda conspired by left‐wing media to magnify the degree to which the Black community is targeted by the police—further discrediting the necessity of anti‐racist movements. The anti‐BLM positions presented so far can also be understood as reinforcing anti‐Black racist stereotypes which portray the Black community as prone to violent, emotional and savage behaviours that the police are obliged to punish. As such, the right‐wing commentators' portrayal of reasonable anti‐Black rhetoric also extends to Black people partaking in BLM demonstrations against the police.

### Constructing BLM protests as violent riots

In this subsection, we highlight how the commentators justify their criticism of BLM‐related demonstrations by using a ‘rioter’ category to reinforce negative stereotypes against Black people. By associating BLM protests with collective acts of violence, the commentators denied the existence of anti‐Black racism while downplaying social justice causes. One way in which the political commentators justified their use of the ‘rioter’ category was by contrasting BLM protesters with civil rights activists who opposed racism in more humane ways—therefore, mitigating against the idea that their anti‐BLM stances are motivated by racism. As an example, consider the following extract where the former state representative candidate—Monique Valenzuela Trudnowski criticizes BLM demonstrations for lacking the same approach to activism against racism. 

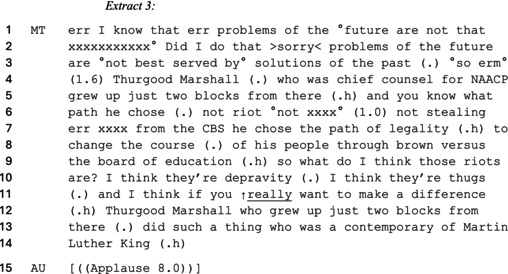

In this account, Trudnowski responds to a question concerning whether BLM demonstrations are legitimate and productive protests. She refutes the idea that BLM demonstrations seek to address and challenge systemic racism against the Black community by presenting these as riots. By using the ‘riot’ category, Trudnowski aligns her argument with the traditional explanation of riots (e.g. Le Bon, 1947) which views the act of participating in them as mindless criminality. Trudnowski does so through additional category work when she describes them as ‘thugs’ (line 10) to portray them as prone to aggressive law breaking. By also accusing them of ‘depravity’ (line 10), Trudnowski cements her depiction of BLM activists as morally reprehensible. By rhetorical contrast, she elevates her position as morally righteous and reasonable—allowing Trudnowski to condemn the BLM movement without making explicitly racist remarks. She thus distances the BLM movement from having any legitimate concern about opposing social injustices (i.e. racism)—as suggested by the social identity framework (e.g. Drury et al., 2020).

To bolster this claim, Trudnowski constructs a distinction between the bad behaviour of the BLM riots and what she depicts as appropriate behaviour of Black people during the civil rights movement. This is done by drawing on the case of Thurgood Marshall of the ‘National Association for the Advancement of Colored People’ (NAACP) civil rights group (note the use of ‘his people’ which positions Black protestors as others by the speaker). By aligning Marshall's approach to activism with ‘legality’, she associates ‘rioting’ with an unjustifiably violent response to anti‐Black racism. In doing so, Trudnowski depicts herself as recognizing past racial inequalities that were necessary to oppose during the civil rights era—as such, distancing her criticism of BLM from racism. Similarly, she also invokes Martin Luther King to strengthen the distinction between the current ‘riots’ which she negatively contrasts with a preferred legal route to campaign. Doing so undermines BLM's anti‐racist message while also presenting its demonstrations as ineffectively addressing social and racial inequalities. By constructing those involved in the BLM protests as immoral and criminal ‘thugs’, Trudnowski draws on and reinforces anti‐Black representations to preserve racism in the United States.

As another example of how the right‐wing commentators used the ‘rioter’ category, consider the next extract where Ben Shapiro dehumanizes BLM protesters for causing unwanted destruction. 

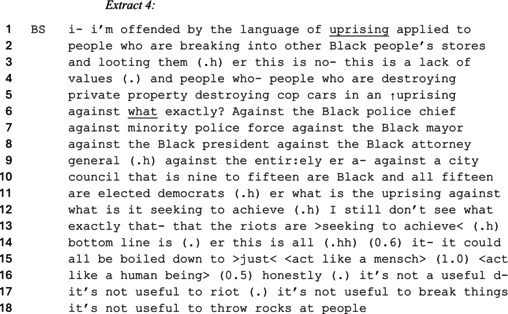

Shapiro undermines the BLM movement by using the ‘rioter’ category on line 13 to associate the actions of the Baltimore protesters with destructive violence (Potter & Reicher, 1987). Edwards (2005) argued that when speakers complain about the consequences of something, they orient towards holding particular people or groups responsible for what happened. Shapiro thus justifies his objection to the ‘riots’ of BLM activists by complaining about them destroying ‘private property’ and ‘cop cars’ (line 5). As indicated by Shapiro's lack of elaboration (see Latour, 1987) concerning why the destruction of these objects constitutes a ‘lack of values’ (3–4), he takes their existence for granted as universal principles representative of civilized US society. Shapiro constructs the destruction of these ideological objects as a defiance of commonsense (Billig, 1987)—justifying his use of the ‘rioter’ category against the Baltimore protesters.

Shapiro argues that the owners of the private property belonged to ‘other Black people’ (line 2) while also indicating that the ‘cop cars’ were used by ‘a minority police force’ (line 7). He presents himself as morally outraged on behalf of the Black community which mitigates the possibility that his anti‐BLM position is motivated by racism. Shapiro poses a rhetorical question: ‘uprising against what exactly?’ (lines 5–6) to frame the BLM demonstration as absurd (Antaki, 2003). By listing and contrasting positions of authority held by Black people (i.e. ‘mayor’, ‘president’ and ‘attorney general’ lines 6–9) with BLM's anti‐racist cause, Shapiro constructs the movement as unnecessary (Jefferson, 1990). When arguing that ‘nine to fifteen’ members of the Baltimore city council are Black and ‘elected Democrats’, he pairs the categories of ‘Black’ and ‘Democrat’. Shapiro therefore treats BLM as self‐defeating by indicating that Black members of the movement are contradicting their group interests by ‘uprising’ against other Black people in power. Doing so substantiates a taken‐for‐granted assumption that anti‐Black racism is a resolved issue of the past (van Dijk, 1992b). Shapiro justifies that the BLM activists lack rationality and are using anti‐racism as an ulterior motive to enact violence—further legitimizing the ‘rioter’ category in association with anti‐Black racist stereotypes.

Shapiro further justifies his use of the ‘rioter’ category by portraying BLM protesters as so deplorable that they must be told to ‘act like a human being’ (lines 15–16). Tileagă (2007, 2015) argued that dehumanizing remarks allow speakers to mitigate that they hold unjustifiable and immoral prejudices against particular groups. Shapiro thus positions members of the BLM demonstrations with animalistic behaviour to justify dehumanizing the Black community. Shapiro strengthens the rationality of his admonishment with the term ‘just’ (line 15) to indicate that civilized conduct is common‐sense behaviour (Lee, 1987). By making morality relevant to the debate, his use of the term ‘mensch’ (line 15) orients to the Yiddish definition—a person of integrity and honour—which he invokes to morally admonish the BLM protests. As a form of rhetorical upgrading, Shapiro replaces the term ‘mensch’ with ‘human being’—reinforcing the trope of animalistic BLM protesters who are incapable of integrity and honour.

In contrast to Shapiro arguing that BLM is unnecessary because members of the Black community hold positions of authority and power, pro‐BLM speakers treated this fact as irrelevant to the existence of racism in the United States (Demasi, 2019). As such, they sought to legitimize the existence of racism against Black people while constructing the movement as peaceful. Like social identity explanations of demonstrations against social injustice, advocates of the movement used a protester category to characterize BLM crowds as legitimizing countermobilizations against anti‐Black racism. As an example, see the final extract where Hawk Newsome claims that he was racially profiled and targeted by the police to justify the necessity of the BLM movement. 

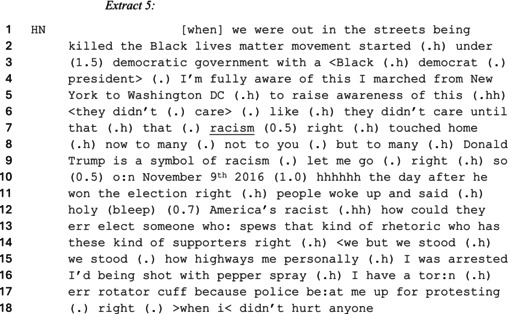

Newsome justifies the legitimacy of the BLM movement by arguing that he was physically assaulted by the police for protesting peacefully. In contrast to Extracts 3 and 4, Newsome uses the ‘protester’ category to describe and justify BLM as a peaceful and non‐violent movement. To legitimize racialized police brutality, he invokes and describes his bodily injuries with specific details when claiming that the police needlessly broke his ‘rotator cuff’ for peacefully protesting (Wiggins & Cromdal, 2021). Newsome also indicates that he has first‐hand experience of being subjected to racialized police brutality. His formulation thus limits the extent to which Owens can refute Newsome's account because, unlike him, she did not witness this event (Wooffitt, 1992). He substantiates the existence of racialized police brutality by depicting the police as violently overreacting to innocuous actions such as protesters standing on highways and not hurting anyone. In doing so, he works up police violence and racism as indistinguishable by indicating that assaulting peaceful Black protesters was unnecessary. Consistent with the social identity explanation of crowds, Newsome's categorical choice of ‘protesting’ (line 17) legitimizes BLM as a response to racialized police violence.

Newsome works up the extent to which the Black community is discriminated against to ward off counterclaims denying the prevalence of racism (see Kirkwood & Goodman, 2018). As such, Newsome on line 3 disclaims that he is oblivious to BLM starting under a ‘Black democrat president’ (lines 3–4) to prevent Owens from using this as evidence against the legitimacy of his anti‐racist position (Hewitt & Stokes, 1975). Doing so contests that the United States can only be legitimately racist under a republican government. Newsome legitimizes the extent to which Black people are discriminated against in the United States despite Barack Obama serving as a ‘Black democrat’ president. Newsome further cements the existence of racialized police brutality by arguing that Black people are ‘in the streets being killed’ (lines 1–2), and the absence of any subjective formulation (e.g. I believe) positions this as a fact independent of his views (Edwards & Potter, 2017). To justify BLM, Newsome frames people as willingly ignoring or overlooking systemic racism in the United States—thus warranting his travel across the nation to raise awareness. Newsome therefore constructs an urgent call for the Black community to mobilize against racialized police brutality.

## DISCUSSION

This study used discursive and rhetorical psychology to explore how anti‐Black stereotypes overlap with anti‐protest arguments when right‐wing political commentators criticized the BLM movement. Like previous discursive research on prejudice, our findings demonstrate that speakers construct themselves as reasonable when endorsing and downplaying anti‐Black racism. We demonstrated that right‐wing commentators denied the racialization of police brutality by treating Black people as predisposed to violent and uncivilized behaviour around the police—therefore, undermining the BLM movement and repeating anti‐Black tropes. In the context of the dataset, the right‐wing commentators’ anti‐Black and anti‐protest rhetoric can be understood as being used interchangeably. Black participants of BLM demonstrations were notably characterized as violent and mindless criminals—criticisms directed towards both crowd members and towards Black people. The ‘uncivilized’ and ‘savage’ behaviour (e.g. Molla, 2024) of crowds which Le Bon claimed to be concerned about was used as rhetoric to criticize the behaviour of Black people who joined BLM protests. Doing so reinforced racist stereotypes associated with them and also the idea that crowd members lose their civilized sense of self. Rather than taking for granted that the right‐wing commentators sought to provide neutral explanations of BLM‐related crowd behaviour, their negative depiction of crowds served an ideological purpose of propagating White supremacist racism.

The findings of the first subsection demonstrated that the political commentators delegitimized the extent to which the Black community is racialized by framing police violence as irrelevant to prejudice. In doing so, the commentators could be understood as using a ‘just‐world argument’ to frame the incidents where Black people were physically assaulted and arrested as natural consequences of *their own* criminal and disorderly behaviour (Goodman & Carr, 2017). The commentators also accused mainstream media of overreporting and exaggerating racialized police violence to construct BLM as a conspiracy that furthers the political stake and interest of the Democratic Party (Byford & Billig, 2001; Edwards & Potter, 1992). Doing so shifted the focus of BLM away from a matter of ‘racists versus anti‐racists’ and instead towards a concern of left‐wing media spreading sinister misinformation about police encounters. The commentators therefore framed their anti‐BLM positions as reasonable to the extent of avoiding the implication that they were predisposed to prejudiced beliefs. In doing so, they reinforced anti‐Black stereotypes by indicating that the Black community is prone to violent criminal tendencies that get them into trouble with the police.

Our findings in the second subsection demonstrated how the right‐wing political commentators depicted those participating in BLM protests as violent—distancing the movement from anti‐racism. We warranted this analytical finding by showing how they used a ‘rioter’ category to frame protests against racialized police brutality as uncivilized and violent. Opponents of BLM presented Black people involved in the demonstrations as mindless and animalistic ‘savages’ to reinforce anti‐Black tropes. As indicated by extract 5, advocates of BLM, in stark contrast to its critics, were more likely to legitimize crowd participation as a response to social injustices like anti‐Black racism. The opponents of the right‐wing commentators therefore framed BLM activists as having legitimate grievances against institutional authorities—as such, their accounts of the demonstrations were similar to social identity theory explanations of crowds. Both sides of the debate used contrasting explanations of BLM crowd behaviour to contest whether Black people participated to mindlessly riot—or to mobilize and protest anti‐Black racism. These contrasting explanations of BLM crowd behaviour are therefore rhetorical resources used by both sides of the debate to challenge or preserve anti‐Black racism.

As indicated by extract 3, the right‐wing commentators rhetorically invoked history to indicate that attempts at countermobilization during the civil rights era were more peaceful and successful than BLM marches. In particular, it is generally characteristic of right‐wing conservatives to treat the actions of Martin Luther King as historical evidence that the civil rights movement solved the systemic issue of racism against Black people in the United States. Throughout the data, the right‐wing commentators avoided mentioning the hardships which Black Americans experienced when fighting for their rights during the civil rights era. With the additional absence of more radical civil rights activists like Malcolm X, the right‐wing commentators strengthened their distinction between uncivilized BLM demonstrations and peaceful protests of the past as a committed historical narrative (see Tileagă, 2018). The ‘rioter’ category therefore functions as a system justifying argument (Hunt, 2025; Hunt et al., forthcoming) which legitimizes a taken‐for‐granted assumption that anti‐Black racism is resolved (van Dijk, 1992b). In doing so, the right‐wing commentators treat BLM and anti‐racist protests as redundant to the extent of suppressing the existence and severity of anti‐Black racism in modern US society.

Our findings highlight a gap in social psychological research, which has not fully considered the similar ways in which Black people and protesters are discriminated against. Historically, Black people and protesters have been the victims of unfair social circumstances and hierarchies. Both groups are notably presented as immoral, uncivilized and violent when opposing their injustice (e.g. Iheme, 2020). Black people are disproportionately subjected to police profiling in addition to being overrepresented in lower socio‐economic positions due to systemic inequalities that restrict opportunities for social mobility (e.g. Black et al., 2006). Given that people join protests due to their frustration with political and economic infrastructures, protesters are often subjected to inequality and prejudice (e.g. Power, 2018). The shared social struggles of Black people and protesters are also magnified by people widely appealing to the universal moral good of ‘law and order’ which can be used, here by right‐wing commentators, to present both Black people and protesters as dangerous threats to social stability. When people fit into both of these groups—Black and a protestor—they are therefore doubly at risk of being presented as immoral, uncivilized and dangerous, which points to an even greater challenge in overcoming anti‐Black racism.

It is important to note, however, that not all activists share the same ethnic identities—many join such protests out of solidarity or a shared experience of oppression (see Drury, 2020). Nevertheless, the ethnic diversity of protesters challenging racial inequalities does not diminish the racist implications of characterizing Black protesters as violent and uncivilized. As a rhetorical strategy, the right‐wing commentators leave the ethnic identities of those participating in BLM protests unspecified. This can work to protect them against being accused of anti‐Black racism for challenging the movement. For another important distinction, Reicher (2024) argues that crowds mobilizing for nefarious purposes, such as intimidating marginalized groups, should not be characterized as protesters—this category is more appropriate for those opposed to social injustice. As such, the anti‐protest rhetoric of right‐wing commentators should not be understood as universally applicable to all political movements—especially not demonstrations endorsing division and inequality. The anti‐protest rhetoric from our data is specifically constructed, situated and oriented towards reinforcing the racist status quo. It is precisely the trope of violent Black young men that makes the pathological characterization of BLM protests racist—particularly when right‐wing anti‐protest rhetoric overlaps with anti‐Black stereotypes.

When right‐wing commentators used the ‘rioter’ category to criticize BLM, it overlaps with racist stereotypes against Black people. They constructed protesters and Black people as uncivilized and overly violent, so it is of note that anti‐protest rhetoric and anti‐Black stereotypes overlap in these particular criticisms of BLM. This means that recognizable anti‐Black stereotypes can be used by those opposed to BLM without overtly presenting violent and uncivilized behaviour as Black characteristics, while still presenting Black people demanding equality in these negative, racialized ways. In focusing on accounts of police brutality against Black people, we addressed Perkins et al.' (in press) call for more discursive studies to focus on anti‐Black racism. The importance of addressing anti‐Black racism is reflected by the right‐wing commentators using two categories concerning ‘violent Black activists’ and ‘uncivilized rioters’ to preserve systemic prejudice against the Black community. The merging of these two categories is a unique finding of this research and points to a rhetorical strategy which anti‐racists need to consider when challenging prejudice. The novelty of this paper demonstrates how the image of a pathological and violent Black BLM protester is a direct consequence of anti‐Black stereotypes overlapping with anti‐protest arguments—coalescing into rhetoric that preserves institutional racism while suppressing dissent.

## AUTHOR CONTRIBUTIONS

**Alexander Hunt:** Methodology; writing – review and editing; writing – original draft; investigation; conceptualization; formal analysis; project administration; data curation. **Mirko Demasi:** Supervision; writing – review and editing; methodology; formal analysis; validation. **Simon Goodman:** Methodology; validation; writing – review and editing; formal analysis.

## CONFLICTS OF INTEREST

The authors declare no conflicts of interest.

## Data Availability

The data analysed in this study were transcribed from two publicly available YouTube videos. The author‐generated transcripts used for analysis are available from the corresponding author upon reasonable request.
